# Quantification of stromal reaction in breast carcinoma and its correlation with tumor grade and free progression survival

**DOI:** 10.1371/journal.pone.0210263

**Published:** 2019-03-21

**Authors:** Xavier Catteau, Philippe Simon, Michel Jondet, Michel Vanhaeverbeek, Jean-Christophe Noël

**Affiliations:** 1 Pathology Department, CUREPATH (CHU Tivoli, Chirec), Jumet, Belgium; 2 Pathology Department, Erasme University Hospital-Université Libre de Bruxelles, Brussels, Belgium; 3 Gynaecologic Department, Erasme University Hospital-Université Libre de Bruxelles, Brussels, Belgium; 4 Gynaecologic Pathology Laboratory, Paris, France; 5 Laboratory of Experimental Medicine, Centre Hospitalo-Universitaire de Charleroi, Montigny-Le-Tilleul, Belgium; University of South Alabama Mitchell Cancer Institute, UNITED STATES

## Abstract

Cancer progression results from a complex interplay between tumor cells and the extracellular milieu. In breast carcinoma, the stromal microenvironment has been suggested to play a major role in promoting tumor growth, progression, and invasion. The stroma of 154 resected specimens of invasive breast carcinoma of no special type was quantified using a digital image analyzer. Statistical analyses were performed between the quantity of stroma and survival, as well as between progression-free survival and clinicopathological data. Levels of myofibroblastic stroma varied from 0–46%, with a median of 15.1% and a standard deviation of 7.5. The myofibroblastic stromal reaction was statistically greater in grade 2 and 3 tumors (p = 0.029). Furthermore, there was a trend for worse progression-free survival in the group of node-negative tumors with strong smooth-muscle actin stromal expression (Log rank = 0.075). The present study demonstrates that the myofibroblastic reaction of breast invasive carcinoma of no special type is not merely a passive reaction, but seems to be an integral part of the neoplastic process by facilitating tumor progression and invasion. Additional, larger studies on mechanisms of stromal change are needed and may potentially lead to novel treatments.

## Introduction

Cancer progression results from a complex interplay between tumor cells and the extracellular milieu. The influence of the microenvironment is of critical importance for the invasiveness of cancer cells [1, 2]. In breast carcinoma, the stromal microenvironment has been suggested to promote tumor growth, progression and invasion [3–5]. Indeed, in a previous study, we showed that peritumoral myofibroblasts expressing smooth-muscle actin (SMA) play a role in ductal carcinoma in situ (DCIS) and invasive carcinoma of no special type (CaNST) [6, 7]. Myofibroblasts are a kind of carcinoma-associated fibroblast (CAF) that modulates the stroma in physiological and pathological conditions through both direct cellular contact and through secretion of different components, such as proteinases, extracellular matrix (ECM) elements, and growth factors [7].

The human eye is limited in the interpretation of histological slides. Hence, computer-aided quantitative digital image analysis (DIA) enables a more complete evaluation of different components [8, 9]. Nevertheless, this technique is not commonly used because it is not widely accessible and is time-consuming.

The aim of the present study was to quantify the tumor stroma in invasive carcinoma of no special type (CaNST) using computer-aided quantitative DIA.

## Materials and methods

### Study population

Breast tumor tissue was retrospectively collected from consecutive patients who were identified through the Pathology Department of Erasme Hospital between July 1997 and November 2002. This study was performed on 154 resected specimens of CaNST. All patients were female. The study protocol was approved by the institutional ethics and research review boards at Erasme Hospital. Patients signed a written informed consent upon admission to the hospital. Patients chose at admission whether to opt in or to opt out of providing consent, which affirms that physicians have the right to use patients’ surplus biological material for research. Consent has been established by the local ethics committee (ethics committee Erasme-ULB, registration number 406) and is in accordance with Belgian and International law (Helsinki declaration).

All archived specimens were reexamined independently by two pathologists (XC, JCN) to confirm the histologic subtype (according to World Health Organization classification). Final pathological tumor stage was determined using the TNM staging system (AJCC Cancer Staging Manual, 8th edition, 2017) and graded using the Nottingham system [10]. For each patient, the following parameters, including patient age, tumor size and grade, TNM classification, lymph node involvement, estrogen receptor (ER) status, progesterone receptor (PR) status, HER2 status and molecular subtypes, were assessed and are summarized in Table 1.

**Table 1:** Clinicopathological data of the study.

		Total cases	
		n	%
Age (years)	<50	45	29.4
	≥50	108	70.6
Tumor size (mm)	≤20	82	53.2
	>20	72	46.8
Lymph node	negative	83	53.9
	positive	71	46.1
Histological grade	grade 1	25	16.3
	grade 2	62	40.6
	grade 3	66	43.1
ER/PR status	negative	16	10.7
	positive	134	89.3
HER2 status	negative	122	81.3
	positive	28	18.7
Molecular subtypes	luminal A	30	20
	luminal B	86	57.3
	luminal HER2	18	12
	HER2 enriched	10	6.7
	triple negative	6	4

Survival data were collected prospectively and included overall survival (OS) and progression-free survival (PFS), defined as the interval from the date of primary treatment to the first distant recurrence, shown in S1 Table.

### Image acquisition

Digitized images of slides were acquired using a Canon 450 EOSD camera. Each case was imaged three or four times at 20X magnification to represent the tumor, and images were saved in JPEG format (3840 x 2820 pixels).

### Image analysis

Initial image analysis was performed using the MAMBA Image analyzer (open-source Mathematical Morphology library).

Segmentation to identify objects of interest was performed by adjusting grayscale data to a binary image with foreground pixels of interest set to black and background pixels set to white. The pixel intensity threshold for classifying all pixels as either foreground or background was automatically defined by the software. Features were extracted by measuring pixel numbers of any particles in the final image. Features extracted included number of stromal SMA-positive cells and total number of cells.

Data points were recorded in pixels and exported to Microsoft Excel alongside clinical outcome data. For each tumor, we obtained the median number of positive stromal SMA cells.

### Immunohistochemistry

Specimens were fixed in histology-grade 4% buffered formalin. A series of paraffin sections were stained with hematoxylin and eosin, and immunohistochemical detection was performed according to the manufacturer’s protocols using a fully automated immunohistochemical system (Autostainer Link 48 from Dako). The SMA antibody was obtained from DAKO (monoclonal mouse, clone 1A4, catalog number IR00611, DAKO, Denmark).

### Statistical analysis

The relationship between DIA patterns of SMA and differential clinical and histological features (age, tumor size and grade, lymph node status, luminal classification) was compared using a Chi-squared test.

Survival curves for OS or PFS were calculated using the Kaplan- Meier method. Different survival curves were compared using the log-rank method.

XLSTAT version 2015.2.01 (Addinsoft) was used for all calculations. In all statistical tests, P< 0.05 was considered significant.

## Results

This study was performed on 154 CaNST, and all patients were female. Age varied from 31–92 years with a mean age of 57 years. Table 1 summarizes the clinical and histological features of the study population.

In all cases, peritumoral stroma appeared fibrous (desmoplastic) upon routine staining with hematoxylin-eosin. Stromal cells were fusiform with spindle-shaped nuclei and did not show nuclear-cytoplasmic atypia.

### DIA stromal SMA expression

**Fig 1:**
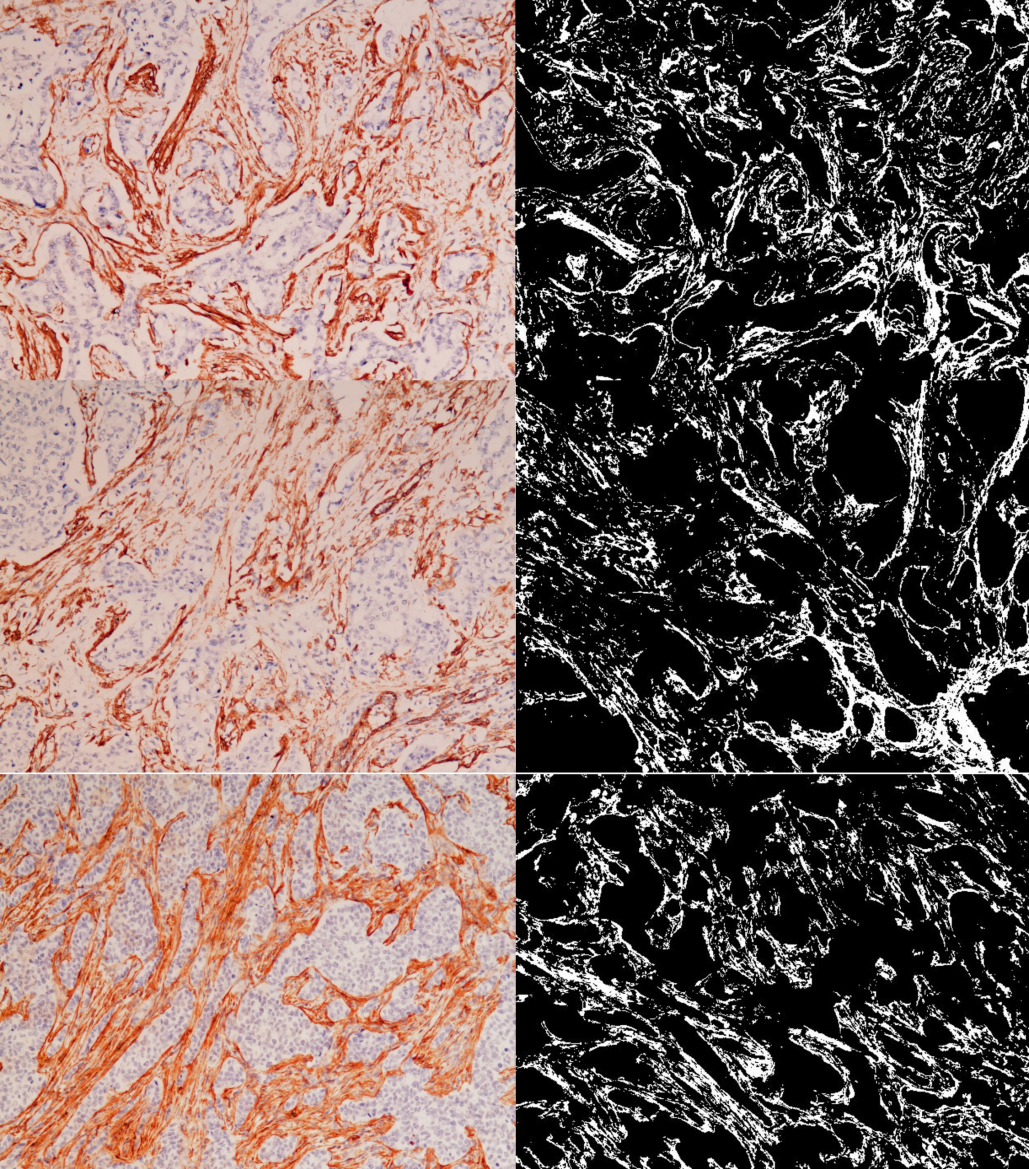
Stromal SMA expression. Immunohistochemical staining of SMA in tumor stroma (left side) and the corresponding image obtained with DIA (right side).

Levels of SMA stroma varied from 0–46%, with a median of 15.1% and a standard deviation of 7.5. Fig 1 shows immunohistochemical staining of SMA in the tumor stroma and the corresponding image obtained with DIA. The myofibroblastic stromal reaction was statistically greater in grade 2 and 3 tumors (p = 0.029). Strong myofibroblastic reaction was found in 51.5, 61.03, and 24% of tumors in grades 3, 2 and 1, respectively. In contrast, no significant relationship was observed between SMA stromal expression and other clinicopathological features. Table 2 summarizes these results.

**Table 2:** Relation of stromal SMA expression and clinicopathological features.

		SMA EXPRESSION			
		>15%	≥5% ≤ 15%		<5%	
Age (years)	<50	51	48	9	p = 0.49
	≥50	26	16	3	
Tumor size (mm)	≤20	48	33	7	p = 0,55
	>20	30	30	5	
Lymph node	Negative	43	35	3	p = 0.11
	Positive	34	27	9	
Histological grade	grade 1	6	15	4	**p = 0.029**
	grade 2	38	20	4	
	grade 3	34	28	4	
ER/PR status	Negative	12	7	1	p = 0.68
	Positive	67	56	11	
HER2 status	Negative	58	54	9	p = 0.29
	Positive	17	8	3	
Molecular subtypes	luminal A	13	14	3	p = 0.57
	luminal B	43	37	5	
	luminal HER2	10	5	3	
	HER2 enriched	7	3	0	
	triple negative	2	3	1	

**Fig 2:**
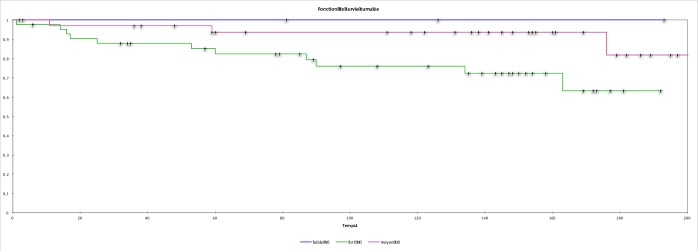
Kaplan-Meier curves for PFS. Kaplan-Meier curves for PFS show a trend for reduced PFS for node-negative tumors with strong myofibroblastic reaction (Log rank: p = 0.075).

There was a trend for worse PFS in the group of node-negative tumors with strong SMA stromal expression (Log rank = 0.075; Fig 2). There was no difference between OS and myofibroblastic reaction.

## Discussion

The importance of changes in the microenvironment during tumor progression is being increasingly recognized [11–13]. We demonstrated in this work the appearance of a myofibroblastic reaction in invasive carcinoma of NST. This reaction is present in almost 100% of cases, irrespective of clinical or histological parameters. This phenomenon is therefore almost ubiquitous and more than likely plays an important tumor role, particularly in the invasive process. Furthermore and importantly, this proinvasive action seems to be confirmed by intense expression of SMA myofibroblasts correlating with the presence of lymph node metastasis [14]. Indeed, myofibroblasts may induce the production of proinvasive proteinases in cancer [15]. In addition to their role in wound healing, myofibroblasts also provide proinvasive signals that in combination affect invasion of cancer cells [16,17]. Cross talk between cancer cells and stromal cells may be mediated through direct heterotypic cell-to-cell contacts or through secreted molecules [18].

Previously, we demonstrated the importance and the constancy of SMA stromal expression in ductal carcinoma in situ (DCIS) and CaNST in a semiquantitative manner [6, 14]. In the present study, we analyzed this myofibroblastic reaction using another method of quantification with the help of DIA. This method allowed us to determine median levels of SMA stroma as 15% in CaNST. To the best of our knowledge, this is the first study that shows quantitative data for the stroma in CaNST. The quantity of stroma was highly variable between different tumors (range 0–46%). Our study also highlighted a statistically significant relationship between myofibroblastic reaction and tumor grade. The myofibroblastic reaction was stronger in higher-grade tumors. This finding could explain a more important secretion of pro-myofibroblastic factors, like TGF-ß, known to be a major player in the SMA stromal reaction, by “more aggressive” tumor cells [14, 19–21].

We observed a trend for reduced PFS in the group of node negative tumors with strong SMA stromal expression. These data suggests that the SMA stromal reaction is a factor conferring poor prognosis in terms of loco-regional recurrence, and this reaction plays a proinvasive role in the neoplastic process.

In conclusion, the present study and our previous work show that the tumoral myofibroblastic reaction in CaNST is not merely a passive reaction but could be an integral part of the neoplastic process by facilitating tumor progression and invasion. Additional, larger studies on mechanisms of stromal change are needed and may potentially lead to novel treatment strategies.

## Supporting information

S1 Table Anonymized patient data. Anonymized data to calculate progression free survival according stromal reaction.
(XLSX)

S2 Table Database SMA survival. (XLSX)
